# Phytochemical Characteristics, Free Radical Scavenging Activities, and Neuroprotection of Five Medicinal Plant Extracts

**DOI:** 10.1155/2012/984295

**Published:** 2011-08-10

**Authors:** Chia Lin Chang, Che San Lin, Guia Hung Lai

**Affiliations:** Research Institute of Biotechnology, HungKuang University, 34 Chung-Chie Rd, Sha Lu, Taichung 43302, Taiwan

## Abstract

The objective of this study was to determine phytochemical characteristics, chemiluminescence antioxidant capacities, and neuroprotective effects on PC12 cells for methanol extracts of *Spatholobus suberectus, Uncaria rhynchophylla, Alpinia officinarum, Drynaria fortunei,* and *Crataegus pinnatifida*. The *C. pinnatifida* extract (CPE) afforded the greatest yield and total phenolic content. The *S. suberectus* extract (SSE) yielded the greatest total flavonoid content. The *U. rhynchophylla* extract (URE) produced the greatest total tannin content, and the *A. officinarum* extract (AOE) produced the greatest total triterpenoid content. The *D. fortunei* extract, assayed using horseradish peroxidase-luminol-hydrogen peroxide (H_2_O_2_), and AOE using pyrogallol-luminol assay each exhibited better antioxidant activity than the L-ascorbic acid and 6-hydroxy-2,5,7,8-tetramethylchroman-2-carboxylic acid did. The CPE, SSE, and URE presented neurogrowth effects and neuroprotective activities on H_2_O_2_-induced PC12 cell death at 0.5–5.0 *μ*g/mL. The CPE represents a promising medicinal plant source for the treatment of H_2_O_2_-induced neurodegenerative disease, because of its useful phytochemical characteristics.

## 1. Introduction

The biological reactivity of free radicals and their roles in oxidative stress are subjects of considerable attention and controversy. Oxygen consumption, necessary for cell growth, generates reactive oxygen species (ROS). The body's normal metabolism, normal use of oxygen, such as respiration, and some cell-mediated immune functions continuously produce ROS. Being active in biological systems, superoxide anion radical (O_2_^∙−^), hydrogen peroxide (H_2_O_2_), hydroxyl radical (^∙^OH), and other reactive oxygen species are collectively referred to as ROS [1, 2]. If cellular agents do not effectively scavenge ROS, then disease will ensue. ROS are implicated in more than 100 diseases [3]. There are specific neurodegenerative diseases that have identified free radical damage as an underlying mechanism, and the rat pheochromocytoma cell (PC12, CRL-1721) model is good to address our research purposes. For example, methyl gallate can significantly scavenge ROS and attenuate the apoptotic response resulting from long-term oxidative stress in H_2_O_2_-induced PC12 cell apoptosis for the neurodegenerative disease study [4]. Sesaminol glucosides have protective effect on beta-amyloid_25–35_ (A*β*_25–35_)-induced PC12 cell death, and its effect may be through the ROS scavenging effect for Alzheimer's disease (AD) study [5]. In PC12 cells, a water extract of the hooks of *Uncaria rhynchophylla* significantly reduces cell death and the generation of ROS induced by 6-hydroxydapamine for Parkinson's disease (PD) study [6]. In addition, Padma 28 is a multicompound herbal preparation, and it has protective effect on the neurotoxicity of PC12 cells induced by the neurotoxins: A*β*, glutamate, 1-methyl-4-phenyl-1,2,3,6-tetrahydropyridine, and 3-nitropropionate, known to be involved in AD, PD, amyotrophic-lateral-sclerosis, and Huntington's disease, respectively. These toxins might have been the result of an oxidative stress which could be attenuated by Padma 28 acting as a potent antioxidant [7].

Currently, natural antioxidants, medicinal plants in particular, are receiving much attention. We had searched related antioxidant references of five medicinal plants (*Spatholobus suberectus*, *U. rhynchophylla*, *Alpinia officinarum*, *Drynaria fortunei*, and *Crataegus pinnatifida*) and found that they had antioxidant activities. We also analyzed antioxidant activity based on the scavenging effect on 1,1-diphenyl-2-picrylhydrazyl radical (DPPH) assay of the methanol extracts for five medicinal plants, and the five methanol extracts presented good DPPH radical scavenging activities (data not shown). These findings led us to believe that the five methanol extracts would be effective in reducing oxidative damage in neural cells. Polyphenols and flavonoids are of interest because of their ability to scavenge ROS. Therefore, it is necessary to characterize the phytochemical properties of plant extracts. Solvent extraction is the most frequently used technique for isolation of plant antioxidant compounds, and alcoholic solutions frequently provide satisfactory results for the extraction process [8]. Chemiluminescence antioxidant assay is a simple, direct, and effective method well suited to free radical and antioxidant study [9]. Therefore, we applied chemiluminescence techniques to assay plant extracts for the ROS scavenging activity. In this study, we investigated the phytochemical characteristics, free radical scavenging effects, and neuroprotective effects of five methanol extracts obtained by ultrasonic extraction in methanol. Samples from each of the five medicinal plants, the methanol extracts were analyzed for four phytochemical characteristics; total phenolic, flavonoid, triterpenoid, and tannin content, using four chemiluminescence antioxidant assays; luminol radical,  ^∙^O_2_^−^, ^∙^OH, and H_2_O_2_, respectively. The PC12 cell line is a useful model for the study of neurodegenerative disease and neuroprotective effects [10]. Thus, we also investigated induced neurogrowth and neuroprotective effects on H_2_O_2_-induced PC12 cell death of the five plant extracts.

## 2. Materials and Methods

### 2.1. Materials

The dried vine stems of *S. suberectus*, the dried hooks of* U. rhynchophylla*, the dried rhizomes of* A. officinarum*, the dried rhizomes of* D. fortunei*, and the dried fruits of *C. pinnatifida* were purchased from Xin Long Pharmaceutical Limited Company (Taichung, Taiwan). Methanol (Mallinckrodt Baker, Inc., Phillipsburg, USA) was purchased as an ACS grade chemical. Deionized water was obtained from an Ultrapure Water System (Putity-UV, Suntex Instruments Corporation, LTD., Taipei, Taiwan). Phosphate buffer (0.1 M, pH 7.4) was prepared from sodium phosphate monobasic monohydrate and sodium phosphate dibasic dodecahydrate. Sodium phosphate monobasic monohydrate, sodium phosphate dibasic dodecahydrate, sodium hydroxide (NaOH), boric acid, gallic acid, glacial acetic acid, perchloric acid, L-ascorbic acid (vitamin C), luminol, horseradish peroxidase (HRP), pyrogallol, sodium bicarbonate (NaHCO_3_), aluminum chloride hexahydrate (AlCl_3_·6H_2_O), vanillin, 3-(4,5-dimethyl-2-thiazolyl)-2,5-diphenyl-2H-tetrazolium bromide (MTT), poly-L-lysine hydrobromide, sodium carbonate (Na_2_CO_3_), ammonia (NH_3_), N-acetyl-Asp-Glu-Val-Asp-al (AC-DEVD-CHO), 1,10-phenanthroline, quercetin, ethylenediaminetetraacetic acid (EDTA), cupric sulfate (CuSO_4_), sodium nitrite (NaNO_2_), zinc acetate (ZnAc), ammonium chloride (NH_4_Cl), and 6-hydroxy-2,5,7,8-tetramethylchroman-2-carboxylic acid (trolox) were originally obtained from Sigma-Aldrich Corporation, Shanghai, China. Trolox and vitamin C were used as positive control samples over an optimized concentration range. Folin-Ciocalteau reagent and ursolic acid were purchased from Fluka Biochemica (Buchs, Switzerland). Thirty-five percent H_2_O_2_ was purchased from Riedel-de Haën (Seelze, Germany). Dimethyl sulphoxide (DMSO) and ethanol were purchased from Merck (Darmstadt, Germany). Dr. Y. C. Shen, of the National Research Institute of Chinese Medicine (Taipei, Taiwan) kindly provided the PC12 cells. Dulbecco's modified Eagle's medium (DMEM), heat-inactivated horse serum (HS), heat-inactivated fetal bovine serum (FBS), penicillin/streptomycin, and L-glutamine were purchased from HyClone (Tseng Hsiang Life Science LTD., Taipei, Taiwan, R.O.C.). The 100 mm cell culture dish was purchased from Greiner Bio-One (Bio-Check Laboratories LTD., Taichung, Taiwan).

### 2.2. Methanol Extraction

The dried vine stems of *S. suberectus*, the dried hooks of* U. rhynchophylla*, the dried rhizomes of* A. officinarum*, the dried rhizomes of* D. fortunei* and the dried fruits of *C. pinnatifida* were pulverized into fine powders using a stainless steel blender (Waring Commercial, Torrington, CT, U.S.A.). Two-gram aliquots of the dried powder were each extracted three-times with methanol (20 mL). The mixtures were agitated in an ultrasonic cleaner (model DC200H, Chemist Scientific Corporation, Taipei, Taiwan, R.O.C.) for 15 min at room temperature then filtered. The methanol filtrates were individually pooled and the methanol solvent removed at 40°C, under reduced pressure by rotary evaporator (Rotavapor R210, Buchi, Postfach, Flawil, Switzerland). Finally, each extract was dried overnight in a freeze dryer (model FD3-12P-80°C, Kingmech Corporation, Taipei, Taiwan, R.O.C.) before calculating the yield of each extract. All of the dried extracts were brown solids, and were stored at −20°C prior to phytochemical characterization and bioassay.

### 2.3. Total Phenolic Content

The total phenolic contents of the five extracts were determined using the Folin-Ciocalteu assay with some modifications [11]. Briefly, 0.6 mg samples of each of the five extracts were dissolved into methanol (1 mL), and then 11.4 *μ*L aliquots of each of these solutions were mixed with Na_2_CO_3_ (2%, 227.3 *μ*L). The solutions were allowed to stand for 2 min at room temperature before adding Folin-Ciocalteau reagent (50%, 11.4 *μ*L) to each sample solution. The mixtures were then incubated for 30 min at room temperature. The absorbances of the reaction mixtures were measured at 750 nm using a tunable microplate reader (VersaMax, Molecular Devices Corporation, Sunnyvale, Calif, U.S.A.). Gallic acid (0.2–1.0 mg/mL in methanol) was used as a standard, and the total phenolic contents of five extracts were expressed in milligram gallic acid equivalents (mg gallic acid/g extract).

### 2.4. Total Flavonoid Content

Flavonoids were determined by the colourimetric method described by Barreira et al. with some modifications [12]. Briefly, 2 mg samples of each of the five extracts were dissolved into methanol (1 mL), and then 25 *μ*L aliquots of each of these solutions were mixed with deionized water (152.5 *μ*L) and NaNO_2_ (5%, 7.5 *μ*L). After 6 min, 15 *μ*L of a 10% AlCl_3_·6H_2_O solution was added, and after a further 5 min incubation at room temperature, 50 *μ*L of 1 M NaOH was added. The tubes were then incubated for 15 min at room temperature. The absorbance at 510 nm was read using a tunable microplate reader. The absorbance of each blank, consisting of the same sample mixtures, but with deionized water in place of 10% AlCl_3_·6H_2_O solution, was subtracted from the test absorbance [13]. Quercetin (0.2–1.0 mg/mL in ethanol) was used as a standard. Results were expressed as milligram quercetin equivalents (mg quercetin/g extract).

### 2.5. Total Triterpenoid Content

After optimizing all experimental parameters, total triterpenoid content was determined by colorimetry using the following procedure [14]. Briefly, 10 mg of each of the five extracts was individually dissolved in 1 mL of methanol. Then, 100 *μ*L of each of these solutions was mixed with vanillin-glacial acetic acid solution (150 *μ*L, 5% w/v) and perchloric acid solution (500 *μ*L). The sample solutions were heated for 45 min at 60°C and then cooled in an ice-water bath to the ambient temperature. After the addition of glacial acetic acid (2.25 mL), each sample solution's absorbance was measured at 548 nm, using a UV-visible-light spectrophotometer (UV-160A; Shimadzu Corporation, Kyoto, Japan). Ursolic acid (0.025–0.5 mg/mL in methanol) was used as a standard. Results were expressed as milligram ursolic acid equivalents (mg ursolic acid/g extract).

### 2.6. Total Tannin Content

Analysis of total tannin was based on a titrimetric method [15]. Zinc ion reacts with tannin compounds in alkali solution, to form complexes. Residual zinc ion is then titrated with EDTA, and zinc complexed tannin is determined from EDTA consumption and total zinc content. The methanol extracts (1 mg each) were each placed into glass vials and dissolved with 1 mL of deionized water. The vials were warmed in a water bath for 5 min at 35 ± 20°C. ZnAc (1 M, 0.4 mL) and NH_3_ (0.28 mL) were mixed together, and the warmed 1-mL extract solutions were added. The solutions were replaced in the water bath for 30 min at 35 ± 2°C. Deionized water (8.92 mL) was added to make the final volume up to 10.6 mL. After careful filtering, sample solutions were obtained. The solutions (0.8 mL) were further diluted with 5.2 mL of deionized water, and 0.5 mL of NH_3_-NH_4_Cl buffer (pH 10) was added. Finally, the mixture was titrated with 0.05 M EDTA. The blank was detected without addition of the extract. The total tannin content (%/mg extract) of each extract was calculated as follows: {[0.1556 × (V_blank_ − V_extract_)]/W_extract_} × 100%. V_blank_ and V_extract_ represent the EDTA titration volumes (mL) recorded for the respective blank and extract solutions. W_extract_ represents the weight (mg) of each extract [16].

### 2.7. HRP-Luminol-H_2_O_2_ System

Samples of 2.5 *μ*L of trolox, vitamin C, five plant extracts, and DMSO as control, were prepared with three replicates, to concentrations of 2.0, 3.9, 7.8, 15.6, 31.3, 62.5, 125.0, 250.0, 500.0, and 1000.0 *μ*g/mL. The samples were mixed with 238.8 *μ*L phosphate buffer (0.1 M, pH 7.4), and 2.6 *μ*L luminol solution (20 mg/mL in DMSO) was added, to yield a final luminol concentration of 1.16 × 10^−3^ M. H_2_O_2_ (1.1 *μ*L) was then added to bring the final concentration to 5 × 10^−2^ M H_2_O_2_. HRP (5.0 *μ*L, 10 IU/mL) was then added to a concentration of 0.2 IU/mL HRP, and a total solution volume of 250 *μ*L. Chemiluminescence was measured with a microplate luminometer apparatus (LUMIstar OPTIMA, BMG labtech GmbH, Offenburg, Germany) for 40 min at 25°C. Integration of the chemiluminescence time-course curves provided an estimate of each sample's relative inhibitory activity under various concentrations. The inhibition ratio (%) of each sample is calculated as follows: [1 − (AUC_sample_/AUC_control_)] × 100%, where AUC_sample_ and AUC_control_ represent the area under the time-course curve measured for the sample solution and control, respectively [17].

### 2.8. Pyrogallol-Luminol System

Samples of 2.5 *μ*L of trolox, vitamin C, five plant extracts, and DMSO as control, were prepared with three replicates, to concentrations of 3.9, 7.8, 15.6, 31.3, 62.5, 125.0, 250.0, 500.0, and 1000.0 *μ*g/mL. The samples were mixed with pyrogallol (6.25 × 10^−4^ M, 12.5 *μ*L), and 235 *μ*L of a mixture containing Na_2_CO_3_-NaHCO_3_ buffer (0.05 M, pH 10.2) with 0.1 mM EDTA/1 mM luminol (2 : 1 v : v) solution was added to yield a final volume of 250 *μ*L. The background was detected without the addition of pyrogallol solution. Chemiluminescence was measured for 10 min at 25°C with a microplate luminometer apparatus. Integration of the sample chemiluminescence time-course curves providing the relative scavenging activity of each sample was estimated at various concentrations. The scavenging activity ratio (%) of each sample was calculated as follows: {[(AUC_control_ − AUC_background_)−(AUC_sample_ − AUC_background_)]/(AUC_control_ − AUC_background_)} × 100%, where AUC_control_, AUC_background_, and AUC_sample_ represent the area under the time-course curve measured for the control, background, and samplfe, respectively [18].

### 2.9. CuSO_4_-Phen-Vc-H_2_O_2_ System

Samples of 12.5 *μ*L of trolox, vitamin C, five plant extracts, and DMSO as control, were prepared with three replicates, to concentrations of 3.9, 7.8, 15.6, 31.3, 62.5, 125.0, 250.0, 500.0, and 1000.0 *μ*g/mL. The samples were mixed with CuSO_4_ solution (1.0 mM, 12.5 *μ*L), 1,10-phenanthroline solution (1.0 mM, 12.5 *μ*L), borate buffer (0.05 M, 175.0 *μ*L, pH 9.0), and L-ascorbate solution (1.0 mM, 25.0 *μ*L). Additionally, H_2_O_2_ solution (12.5 *μ*L, 0.15% v/v) was added to yield a final volume of 237.5 *μ*L. The background was detected without the addition of H_2_O_2_ solution. Chemiluminescence was measured for 10 min at 25°C with a microplate luminometer apparatus. The scavenging activity ratio (%) of each sample was calculated using the formula described for the pyrogallol-luminol system [18].

### 2.10. Luminol-H_2_O_2_ System

Samples of 12.5 *μ*L of trolox, vitamin C, five plant extracts, and DMSO as control, were prepared with three replicates, to concentrations of 3.9, 7.8, 15.6, 31.3, 62.5, 125.0, 250.0, 500.0, and 1000.0 *μ*g/mL. The samples were mixed with H_2_O_2_ solution (0.15 M, 12.5 *μ*L). A solution containing Na_2_CO_3_-NaHCO_3_ buffer solution (225 *μ*L, 0.05 M, pH 9.4) and luminol solution (0.1 mM, 17 : 1 v : v) was added to yield a final volume of 250.0 *μ*L. The background was detected without the addition of H_2_O_2_ solution. Chemiluminescence was measured for 40 min at 25°C with a microplate luminometer apparatus. The scavenging activity ratio (%) of each sample was calculated using the formula described for the pyrogallol-luminol system [18].

### 2.11. Assay for PC12 Cell Viability and Protective Effect

PC12 cells were plated on poly-L-lysine hydrobromide-coated 100 mm cell culture dishes, grown in DMEM, and supplemented with 10% HS, 1% FBS, a mixture of 1% penicillin/streptomycin and 1% L-glutamine at 37°C in a 95% humidified air-5% CO_2_ chamber. Cells were subcultured for up to ten passages. Cellular viability was determined using the trypan blue exclusion test. Only cell preparations with 95% or greater viability were used. PC12 cells were seeded in poly-L-lysine hydrobromide-coated 24-well cell culture plates (1.25 × 10^5^ cells/well) with complete DMEM for 24 h. Thereafter, the PC12 cell viability and protective effect assays were completed by the following procedure: (1) the media were replaced with fresh media, and cells were treated with or without the presence of 500 *μ*L test samples to provide sample concentrations of 0, 0.25, 0.5, 2.5, and 5.0 *μ*g/mL. The cultures were then incubated at 37°C in a 95% humidified air-5% CO_2_ chamber for various periods (12, 24, 48, and 72 h). The control was incubated without the addition of the sample solution. (2) H_2_O_2_ was added to induce PC12 cell death. To study the protective effect of test samples on the PC12 cells, we renewed the media and preincubated the cells for 12 h, either with or without the presence of 500 *μ*L test samples, to obtain sample concentrations of 0, 0.5, 2.5, and 5.0 *μ*g/mL. Thereafter, the media were replaced, and 500 *μ*L H_2_O_2_ was added to a concentration of 40 *μ*M H_2_O_2_, and the mixture incubated for an additional 12 h. The control was incubated without the addition of either sample or H_2_O_2_ solution.

All extracts were dissolved in DMSO. The concentration of DMSO in the final culture medium was 0.5%, which had no observable effect on cell viability as determined by MTT reduction assay, following Choi et al.'s method with slight modifications [19]. Upon completion of incubation, MTT solution (50 *μ*L, 1.0 mg/mL) was added to the culture medium, and the cells were incubated for 2 h at 37°C. The medium was then removed, and 300 *μ*L of DMSO was added to the well to dissolve the formazan, derived from live cell mitochondrial cleavage of the tetrazolium ring. The formazan solutions were incubated for 30 min at 25°C, and formazan solutions (250 *μ*L) were placed in 96-well plates. The amount of MTT formazan product was determined by measuring optical density (OD) with a tunable microplate reader at a test wavelength of 570 nm and a reference wavelength of 655 nm. MTT reduction was calculated as OD_570 nm_ − OD_655 nm_. Cell viability (%) was determined as (OD_sample_/OD_control_) × 100%, where OD_sample_ represents OD_sample(570 nm)_ − OD_sample(655 nm)_ and OD_control_ represents the mean value of OD_control(570 nm)_ − OD_control(655 nm)_. The control cells exhibited 100% cell viability. AC-DEVD-CHO, a caspase-3 inhibitor, was the positive control.

### 2.12. Statistical Analysis

The IC_50_ values (the concentration of a sample that is required for 50% inhibition *in vitro*) were determined using linear regression. Each phytochemical characteristic and chemiluminescence antioxidant activity was determined three times, using the same extract in order to determine reproducibility and to provide a mean ± standard deviation (SD) using Microsoft Excel 2003. Cell viability was measured by MTT reduction assay. Cell viability (%) represents three replicates per treatment. For each sample's “cell viability” towards PC12 cells, only data concerning PC12 cell group exposure to each sample were considered. For each sample's “protective effect” against H_2_O_2_ effects on PC12 cells, only data concerning PC12 cell group exposure to H_2_O_2_  and each sample were considered. All data were represented as means ± SD based on triplicate determinations. Data were analyzed for statistical significance using one-way ANOVA, followed by Tukey's test as a *post hoc* test with SPSS software (SPSS for Windows, Version 10).

## 3. Results and Discussion

### 3.1. Extraction Yield and Total Phenolic, Flavonoid, Triterpenoid, and Tannin Content of Methanol Extracts

Table 1 presents the yield and total phenolic, flavonoid, triterpenoid, and tannin content of the five methanol extracts. The yield of the five extracts varied from 2.8 to 22.0%. The *C. pinnatifida *extract (CPE) had the greatest yield of the five extracts. The *U. rhynchophylla* extract (URE) produced the lowest extraction yield of all samples analyzed.

Total phenolic content in the five extracts was determined from a linear gallic acid standard curve. The total phenolic content of five extracts varied from 241.7 to 599.0 mg gallic acid/g extract. This result suggests that the CPE provided the greatest concentration of phenolic compounds of the five plants. The total flavonoid content of five extracts was evaluated by the aluminum colorimetric method, using quercetin as the standard. The total flavonoid content of five extracts varied considerably from 393.0 to 1137.4 mg quercetin/g extract. This result indicates that the *S. suberectus* extract (SSE) was richest of all samples in flavonoids. The total triterpenoid content of the five extracts was evaluated by colorimetry, using ursolic acid as the standard. The total triterpenoid content of five extracts varied from 1.7 to 109.8 mg ursolic acid/g extract. The lowest total triterpenoid content occurred for the CPE, whereas *A. officinarum *extract (AOE) provided the highest triterpenoid content of the group. The total tannin content of five extracts varied from 13.7 to 57.0%/mg extract. The highest content of total tannin was detected in the URE. Finally, these results show that the CPE produced both the best extraction yield and largest total phenolic content but had the lowest total triterpenoid content of the five plant extracts. The SSE produced the greatest total flavonoid, content, and the AOE had the greatest total triterpenoid content. The URE produced the lowest extraction yield, though having the greatest total tannin content. However, the *D. fortunei* extract (DFE) had the lowest total phenolic, flavonoid and tannin content of all samples.

### 3.2. Antioxidant Activity of the HRP-Luminol-H_2_O_2_ System

The antioxidant activity of five extracts was evaluated using a chemiluminescence assay method (Table 2). The HRP-luminol-H_2_O_2_ system can markedly amplify light emission. The scavenging effect of luminol radical was observed upon addition of the five extracts. The IC_50_ values for the five extracts' antioxidant activities ranged from 2.9 to 544.2 *μ*g/mL. The antioxidant abilities of the five extracts compared with vitamin C and trolox were in the order DFE > trolox > SSE > URE > vitamin C > AOE > CPE. However, this finding shows that the luminol radical scavenging effect of the DFE is unrelated to the amount of total phenolic, flavonoid, triterpenoidm, and tannin content.

### 3.3. Antioxidant Activity of the Pyrogallol-Luminol System

The antioxidant activity of five extracts was evaluated by pyrogallol-luminol assay (Table 2). Pyrogallol was autoxidized under alkaline conditions to generate ^∙^O_2_^−^, and the scavenging effect of ^∙^O_2_^−^ was observed on addition of each of the five extracts. The antioxidant properties of the five extracts, against both vitamin C and trolox, were in the order of AOE > trolox > CPE > URE > vitamin C/SSE/DFE, demonstrating the different antioxidant properties of the five extracts. The greater ^∙^O_2_^−^ scavenging effect exhibited by the AOE relates to the amount of total triterpenoid content.

### 3.4. Antioxidant Activity of the CuSO_4_-Phen-Vc-H_2_O_2_ System

The antioxidant activity of five extracts was evaluated by the CuSO_4_-Phen-Vc-H_2_O_2_ assay method (Table 2). The ^∙^OH scavenging effect was observed by adding each of the five extracts. The IC_50_ values for the five extracts' antioxidant activity ranged from 5.9 to 27.7 *μ*g/mL. The antioxidant properties of the five extracts against both vitamin C and trolox were in the order trolox > URE > AOE > CPE > SSE > DFE > vitamin C, indicating the varying ^∙^OH scavenging activities of the five extracts. The URE's high ^∙^OH scavenging activity results from its total tannin content.

### 3.5. Antioxidant Activity of the Luminol-H_2_O_2_ System

The antioxidant activity of five extracts was evaluated using luminol-H_2_O_2_ assay (Table 2). In the oxygen and alkaline solution, H_2_O_2_ oxidizes luminol to produce luminescence. The five extracts' IC_50_ values for scavenging H_2_O_2 _ranged from 1.1 to 9.2 *μ*g/mL. The antioxidant properties of the five extracts, together with vitamin C and trolox, were in the decreasing order of activity: trolox > CPE > SSE > URE > AOE > DFE > vitamin C. Thus, all five extracts were able to scavenge H_2_O_2_. The best H_2_O_2_ scavenging activities, by CPE and SSE, relate to the amounts of total phenolic and flavonoid content in their extracts, respectively.

### 3.6. PC12 Cell Viability and Protection Against Oxidative Damage *In Vitro*

PC12 cells were incubated with various sample concentrations (0.25, 0.5, 2.5, and 5.0 *μ*g/mL) for 12, 24, 48, and 72 h.  Table 3 compares sample-induced neurogrowth effects, evaluated by MTT reduction assay, for the five extracts together with values for the AC-DEVD-CHO positive control. The greatest neurogrowth activities among the five extracts occur at 12 h, for SSE, URE, and CPE. The SSE still showed good neurogrowth activity at 24–72 h. The URE, did not exhibit good neurogrowth activity during the 24–72 h period, and the CPE did not have good neurogrowth activity at 72 h. However, AOE and DFE produce neither good nor stable neurogrowth activities in the 12–72 h period. These results indicate that SSE, URE and CPE produce similar neurogrowth effects. This observed stimulation in neurogrowth activity may result from these three extracts' antioxidant properties. 

In order to ascertain the neuroprotective effects of the five extracts, we used an *in vitro* “Inhibition of H_2_O_2_-induced PC12 cell death” model to estimate the neuroprotective effect. In our study, we found that cell viability decreased to 59.0 ± 5.1% when exposed to H_2_O_2_  (40 *μ*M, 12 h), as shown in Table 4 [20]. Table 4 also compares neuroprotective effects of the five extracts with the positive control, AC-DEVD-CHO. The control showed the highest neurogrowth activity but did not provide the PC12 cells with protection against H_2_O_2_ solution. The CPE, SSE, and URE inhibited H_2_O_2_-induced cytotoxicity at 0.5–5.0 *μ*g/mL. The neuroprotective effects provided by these three extracts may be the reason for the observed stimulation of neurogrowth activity. Table 4 shows that protective properties of the three extracts are in the order CPE > URE > SSE. The CPE exhibited the greatest neuroprotective activity among the five extracts, and the AOE and DFE did not provide any observable neuroprotective effects. The effective neuroprotective activity of the CPE is a consequence of its ^∙^O_2_^−^ ion and H_2_O_2_ scavenging activities, its high extraction yield, and its total phenolic content. The neuroprotective property of the URE is due to the extract's luminol radical and ^∙^OH scavenging activities, and the high total tannin content. The neuroprotective activity of the SSE is a consequence of its luminol radical and H_2_O_2_ scavenging activities and to its total flavonoid content.

## 4. Conclusions

We demonstrated and compared for the first time the phytochemical characteristics and chemiluminescence antioxidant activities of five medicinal plant extracts. The methanol extracts provided protection to PC12 cells against oxidative stress *in vitro*. The SSE had the highest total flavonoid content and exhibited the greatest antioxidant activities in the HRP-luminol-H_2_O_2_ and luminol-H_2_O_2_ assays. The URE appeared to have high total tannin content and showed good antioxidant activities in both HRP-luminol-H_2_O_2_ and CuSO_4_-Phen-Vc-H_2_O_2_ assays. The AOE exhibited high total triterpenoid content and good antioxidant activities for the pyrogallol-luminol and CuSO_4_-Phen-Vc-H_2_O_2_ assays. The DFE had good antioxidant activity in the HRP-luminol-H_2_O_2_ assay. The CPE had the highest total phenolic content, with good antioxidant activities in the pyrogallol-luminol and luminol-H_2_O_2_ assays. Thus, the five extracts present various levels of ROS scavenging efficiency due to differences between the mechanisms of the four ROS chemiluminescence systems. The CPE, SSE, and URE exhibit neurogrowth effects and neuroprotective activities against H_2_O_2_-induced toxicity toward PC12 cells. The five extracts are new potential sources of natural antioxidants for food and nutraceutical products. The CPE is a potential candidate for application in treatment of H_2_O_2_-induced neurodegenerative disease. Therefore, AD and PD diseases that are in part due to ROS may potentially be thwarted by the CPE. Further investigations are necessary to verify these extracts' activities *in vivo*.

## Figures and Tables

**Table 1:** Extraction yield and total phenolic, flavonoid, triterpenoid, and tannin content of the methanol extracts for five medicinal plants.

Medicinal name	Extraction yield^a^	Total phenolic content^b^	Total flavonoid content^c^	Total triterpenoid content^d^	Total tannin content^e^
*Spatholobus suberectus*	4.6 ± 0.5	532.6 ± 15.5	1137.4 ± 15.6	45.7 ± 1.4	42.6 ± 1.4
*Uncaria rhynchophylla*	2.8 ± 0.2	242.7 ± 8.4	410.9 ± 7.6	61.3 ± 3.7	57.0 ± 2.2
*Alpinia officinarum*	4.8 ± 0.3	376.8 ± 5.5	659.5 ± 10.4	109.8 ± 5.6	45.4 ± 1.2
*Drynaria fortunei*	5.8 ± 0.4	241.7 ± 6.5	393.0 ± 2.2	35.2 ± 2.4	13.7 ± 0.8
*Crataegus pinnatifida*	22.0 ± 1.1	599.0 ± 16.0	472.4 ± 11.5	1.7 ± 0.2	35.0 ± 3.6

The data are presented as mean ± SD for three replicates.

^a^% w/w.

^b^mg gallic acid/g extract.

^c^mg quercetin/g extract.

^d^mg ursolic acid/g extract.

^e^%/mg extract.

**Table 2:** Antioxidant activities of the methanol extracts for five medicinal plants and the positive controls.

Medicinal name and positive control	HRP-luminol-H_2_O_2_ assay^a^	Pyrogallol-luminol assay^a^	CuSO_4_-Phen-Vc-H_2_O_2_ assay^a^	Luminol-H_2_O_2_ assay^a^
Medicinal name				
*Spatholobus suberectus*	3.3 ± 0.6	>1000	17.8 ± 1.6	1.3 ± 0.3
*Uncaria rhynchophylla*	3.6 ± 0.4	474.6 ± 15.1	5.9 ± 0.5	2.0 ± 0.5
*Alpinia officinarum*	47.0 ± 2.4	77.1 ± 7.5	8.3 ± 0.3	2.3 ± 0.4
*Drynaria fortunei*	2.9 ± 0.3	>1000	27.7 ± 1.0	9.2 ± 2.5
*Crataegus pinnatifida*	544.2 ± 21.8	328.6 ± 10.2	13.2 ± 0.6	1.1 ± 0.1

Positive control				
Vitamin C	14.8 ± 6.2	>1000	688.3 ± 29.7	32.8 ± 8.9
Trolox	3.2 ± 0.1	209.6 ± 17.0	5.8 ± 1.0	0.8 ± 0.1

The data are presented as mean ± SD for three replicates.

^a^IC_50_ values in *μ*g/mL.

**Table 3:** Effects of the methanol extracts for five medicinal plants and positive control on PC12 cell viability.

Sample	Concentration (*μ*g/mL)	Cell viability (%)			
		12 hr	24 hr	48 hr	72 hr
Control	0	100.0 ± 11.9	100.0 ± 13.8	100 ± 14.8	100.0 ± 13.9

*Spatholobus suberectus*	0.25	102.0 ± 13.3	86.3 ± 12.8	102.0 ± 15.3	136.1 ± 15.8*
	0.5	174.4 ± 21.0***	122.9 ± 16.2	128.1 ± 12.0	208.0 ± 5.3***
	2.5	125.8 ± 12.2	106.8 ± 4.9	136.6 ± 4.2*	113.8 ± 11.9
	5.0	148.0 ± 8.9**	203.5 ± 22.3***	169.9 ± 17.3***	146.1 ± 15.3**

*Uncaria rhynchophylla*	0.25	99.6 ± 3.1	45.2 ± 5.0***	73.8 ± 4.2*	60.8 ± 1.8***
	0.5	110.6 ± 9.9	59.4 ± 4.5***	87.4 ± 2.1	73.3 ± 7.0*
	2.5	93.3 ± 15.6	69.4 ± 8.3**	72.4 ± 10.7*	57.4 ± 1.8***
	5.0	144.9 ± 6.4***	82.5 ± 10.3	64.7 ± 0.5***	57.6 ± 6.2***

*Alpinia officinarum*	0.25	77.6 ± 8.1	79.7 ± 9.3	78.9 ± 14.6	66.5 ± 1.5***
	0.5	102.3 ± 15.7	54.0 ± 3.0***	69.4 ± 2.7	77.0 ± 7.9
	2.5	50.0 ± 9.7***	69.3 ± 3.6*	101.1 ± 15.2	39.4 ± 3.7***
	5.0	89.3 ± 7.9	78.8 ± 11.3	82.0 ± 12.3	54.2 ± 6.7***

*Drynaria fortunei*	0.25	60.7 ± 8.7***	36.1 ± 1.9***	39.6 ± 5.3***	22.4 ± 0.8***
	0.5	60.0 ± 9.4***	37.5 ± 5.2***	44.2 ± 1.4***	35.1 ± 6.4***
	2.5	81.8 ± 7.7	35.4 ± 3.5***	49.4 ± 5.7***	36.9 ± 2.9***
	5.0	85.4 ± 6.4	35.7 ± 4.4***	49.0 ± 6.8***	25.4 ± 4.1***

*Crataegus pinnatifida*	0.25	60.7 ± 4.5**	79.7 ± 10.3	95.4 ± 5.7	44.3 ± 4.2***
	0.5	77.9 ± 4.1	53.9 ± 3.0***	117.1 ± 1.7	64.2 ± 8.0**
	2.5	105.9 ± 17.2	69.3 ± 3.6*	109.9 ± 9.1	27.1 ± 5.2***
	5.0	171.6 ± 9.3***	98.1 ± 8.1	111.1 ± 18.2	56.6 ± 9.4***

AC-DEVD-CHO	0.25	170.5 ± 23.8***	97.0 ± 16.7	95.5 ± 11.4	173.6 ± 27.7***
	0.5	76.9 ± 4.5	100.0 ± 12.0	99.1 ± 8.8	229.6 ± 17.0***
	2.5	222.0 ± 16.7***	120.2 ± 8.9	294.7 ± 26.6***	204.1 ± 15.5***
	5.0	189.3 ± 22.1***	129.0 ± 23.9	190.2 ± 22.3***	191.1 ± 19.1***

The data are presented as mean ± SD for three replicates.  *P < 0.05, **P < 0.01, ***P < 0.001 versus the control group without the addition of the sample solution.

**Table 4:** Protective effects of the methanol extracts for five medicinal plants and positive control on H_2_O_2_-induced PC12 cell death.

Sample	Concentration (*μ*g/mL)	Cell viability (%)
Control	0	100.0 ± 7.3

H_2_O_2_	40 *μ*M	59.0 ± 5.1^#^

*Spatholobus suberectus*	0.5	46.3 ± 3.1
	2.5	67.4 ± 2.9
	5.0	65.7 ± 7.9

*Uncaria rhynchophylla*	0.5	54.1 ± 1.6
	2.5	61.0 ± 6.6
	5.0	85.7 ± 5.6*

*Alpinia officinarum*	0.5	15.4 ± 3.0**
	2.5	13.0 ± 1.2**
	5.0	14.5 ± 2.8**

*Drynaria fortunei*	0.5	17.0 ± 1.5**
	2.5	16.0 ± 0.9**
	5.0	19.8 ± 1.5**

*Crataegus pinnatifida*	0.5	78.1 ± 10.0
	2.5	83.4 ± 3.0*
	5.0	107.2 ± 6.4**

AC-DEVD-CHO	0.5	23.9 ± 4.2**
	2.5	19.7 ± 3.3**
	5.0	37.5 ± 5.8

The data are presented as mean ± SD for three replicates.  ^#^ *P* < 0.01 versus the control group without the addition of the sample and H_2_O_2_ solutions. *P < 0.05; **P < 0.01 versus the 40 *μ*M H_2_O_2_-treated group without the addition of the sample solution.
